# Revealing the nuclearity of iron citrate complexes at biologically relevant conditions

**DOI:** 10.1007/s10534-023-00562-1

**Published:** 2023-12-18

**Authors:** Maria Gracheva, Zoltán Klencsár, Zoltán Homonnay, Ádám Solti, László Péter, Libor Machala, Petr Novak, Krisztina Kovács

**Affiliations:** 1 Department of Analytical Chemistry, Institute of Chemistry, ELTE Eötvös Loránd University, Pázmány P. s. 1/A, 1117 Budapest, Hungary; 2 Nuclear Analysis and Radiography Department, Centre for Energy Research, Konkoly-Thege Miklós út. 29-33, 1121 Budapest, Hungary; 3 Department of Plant Physiology and Molecular Plant Biology, Institute of Biology, ELTE Eötvös Loránd University, Pázmány Péter s. 1/C, 1117 Budapest, Hungary; 4 Department of Complex Fluids, Institute for Solid State Physics and Optics, Wigner Research Centre for Physics, Konkoly-Thege Miklós út 29-33, 1121 Budapest, Hungary; 5 Department of Experimental Physics, Faculty of Science, Palacký University Olomouc, 17. listopadu 1192/12, 771 46 Olomouc, Czech Republic

**Keywords:** Iron, Citrate ligands, Mössbauer spectroscopy, EPR, Iron metabolism

## Abstract

Citric acid plays an ubiquitous role in the complexation of essential metals like iron and thus it has a key function making them biologically available. For this, iron(III) citrate complexes are considered among the most significant coordinated forms of ferric iron that take place in biochemical processes of all living organisms. Although these systems hold great biological relevance, their coordination chemistry has not been fully elucidated yet. The current study aimed to investigate the speciation of iron(III) citrate using Mössbauer and electron paramagnetic resonance spectroscopies. Our aim was to gain insights into the structure and nuclearity of the complexes depending on the pH and iron to citrate ratio. By applying the frozen solution technique, the results obtained directly reflect the iron speciation present in the aqueous solution. At 1:1 iron:citrate molar ratio, polynuclear species prevailed forming most probably a trinuclear structure. In the case of citrate excess, the coexistence of several monoiron species with different coordination environments was confirmed. The stability of the polynuclear complexes was checked in the presence of organic solvents.

**Supplementary Information:**

The online version contains supplementary material available at 10.1007/s10534-023-00562-1.

## Introduction

Iron is considered as one of the most important microelement in nature. It has a key role in the enzymatic processes of bacteria, as well as of plants and mammals. However, because of the low solubility of ferric ion at physiological conditions, the complexation of iron(III) has a crucial role in the iron uptake and transport of living organisms. Citric acid is a well-known natural compound which is able to form stable complexes with iron(III). The resulting iron(III) citrate system consists of a diverse group of ferric carboxylate complexes containing ferric ions and citrate ligands exhibiting different levels of protonation. The involvement of these complexes in the iron metabolism is of significant importance: citric acid is known to facilitate the bioavailability of iron and enhance Fe transport through the cell membranes (Knutson 2019; Kobayashi et al. 2019). Among bacteria, *Escherichia coli* cells utilise multiple iron citrate complexes in their iron uptake processes (Braun and Herrmann 2007; Banerjee et al. 2016). The root-to-shoot transport of iron in the xylem of plants was shown to depend on citrate (Terzano et al. 2013; Ariga et al. 2014). Authors of (Rellán-Álvarez et al. 2010) showed that among the possible iron citrate complexes the formation of tri-iron(III), tri-citrate complexes are favoured at high Fe-to-citrate molar ratio in xylem sap of tomato (*Solanum lycopersicum*), whereas binuclear iron citrate species such as di-iron(III), di-citrate are preferred to be formed in lower Fe-to-citrate environment in *Lycopersicum esculentum* (Ariga et al. 2014). Nevertheless, taxon-specific differences were suggested among plant species (Ariga et al. 2014), and subsequently monomers were detected in the xylem sap of *Cucumis sativus* (Singh et al. 2023). Since not only the secretion of citrate (Durrett et al. 2007) but the oxidation of iron (Xu et al. 2022) stays under a biological control, the formation of iron citrate complexes is affected by the plant organisms. Although the information on the chemical form of the labile iron in the cells is limited (Sági-Kazár et al. 2022), the preference of chloroplasts towards complexes with 1:1 iron(III) to citrate stoichiometry suggests the role of citrate in the intracellular ligation of iron (Müller et al. 2019). In mammals, citrate is among the most important ligands of iron. It can be found in the blood plasma and functions as one of the major complexation agent of non-transferrin-bound iron (Knutson 2019; Evans et al. 2008). In medicine, iron(III) citrate was approved in the European Union and in the United States for improving hemoglobin response and iron parameters for adult patients with iron deficiency anemia and chronic kidney disease (Gupta et al. 2018).

In spite of their biological importance, the precise speciation and coordination chemistry of ferric citrate in aqueous solutions are still not well understood (Pierre and Gautier-Luneau 2000; West et al. 2023). Until now, altogether six iron(III) citrate complexes have been structurally characterized in the solid state, each displaying a distinct mode of coordination of the citrate ligand(s) (Gautier-Luneau et al. 2005). Nevertheless, the determination of metal citrate speciation in aqueous environments continues to pose challenges and remains largely uncertain (Bodor et al. 2002). Significant discrepancies and contradictory findings have been documented in the scientific literature regarding the speciation of iron citrate under various conditions (concentrations and pH values). Multiple structures differing in iron nuclearities, ratios of iron to citrate, and ligand coordination modes were proposed by different authors (Shweky et al. 1994; Matzapetakis et al. 1998; Bino et al. 1998; Silva et al. 2009a; Ganz et al. 2019). Recently, using mass spectrometry it was shown that the monoiron dicitrate (Fe(Cit)_2_)^5−^ complex and polynuclear species of low nuclearity can coexist in the solution at certain conditions (Silva et al. 2009a). Special attention should be devoted to the Fe citrate speciation in solutions at physiological conditions. The leaf xylem sap pH and iron-to-citrate ratio can change in response to variation in environmental conditions experienced by the root (such as soil drying or flooding, exposure to salts, etc.) but normally its pH value is close to 5.5 (Grunwald et al. 2021). The pH values in the blood plasma lies mostly in the neutral pH region, while the approximate physiological ratio of iron-to-citrate was found to be 1:100 (Dziuba et al. 2018).

To get further information on the stability of the polynuclear structure of iron complexes, additional organic solvent (dimethyl sulfoxide, DMSO) showing high coordination ability to Fe^3+^ was applied to Fe^3+^ citrate system, and for comparison, also for the Fe^3+^-EDTA complex. Both complexes are widely applied and compared as a source of iron for biological experiments (Botebol et al. 2014). DMSO was shown to perform a rapid penetration of substances across biologic membranes (Brayton 1986) and low toxicity for cells in vitro (Da Violante et al. 2002). Therefore, DMSO − water mixtures are widely used in biological studies serving as a proper solvent for several biomolecules such as flavins, carotene, etc. (Akladios et al. 2016; Rajniak et al. 2018; Tsai et al. 2018). However, the possibility of iron complexation by the solvent ligands is normally not considered in these studies.

Among the various spectroscopic techniques commonly utilized for the characterization of iron compounds, ^57^Fe Mössbauer spectroscopy plays a special role. In solid materials, it enables the detection of iron with any electronic configuration, independent of oxidation and spin states, offering both qualitative and quantitative insights (Nakajima et al. 2019; Kamnev et al. 2004; Merkel et al. 2008). Although being a method usually applied for solid state physics, Mössbauer spectroscopy presents also an effective approach for investigating the chemical structures of solutions, if applied in a frozen state (Vértes and Nagy 1990). Rapid cooling may preserve the coordination and bonding conditions of the dissolved species in the solution (Ruby et al. 1968), thereby ^57^Fe Mössbauer spectroscopy can provide reliable data regarding the oxidation state and chemical speciation of iron in the original solution, including quantitative information on relative occurrences of mono- and polynuclear structures of solvates.

In addition, application of electron paramagnetic resonance (EPR) spectroscopy in conjunction with Mössbauer spectroscopy may provide further insights into the iron(III) states present in the frozen solution samples. Results obtained with EPR may also help the accurate analysis of Mössbauer spectra through the common application of the theoretical concept of spin Hamiltonian formalism (Oosterhuis 1974).

## Materials and methods

### Synthesis

A stock solution containing enriched ferric chloride ^57^FeCl_3_ (enriched in ^57^Fe, ca. 90%) was prepared and subjected to Mössbauer spectroscopy analysis prior to further preparations. Citric acid monohydrate (> 99.7% purity, VWR Chemicals, BDH Prolabo, Belgium) served as the source of citrate. To reveal iron speciation, solutions with Fe to citrate molar ratios ranging from 1:1 to 1:100 were prepared at physiologically relevant pH values: 5.5 (xylem sap) and 7.0 (cell cytoplasm). Throughout the preparation process, the pH of the solutions was carefully maintained within the desired range using appropriate portions of KOH solutions of various concentrations. The final iron concentration of solutions was 10 mM, unless explicitly given otherwise.

### Mössbauer spectroscopy

Following preparation, the solutions were quickly (drop wisely) frozen on the surface of a metal slab immersed in liquid nitrogen and placed in a liquid nitrogen bath type cryostat. ^57^Fe Mössbauer spectroscopy measurements were carried out on the frozen solutions at *T* = 80 K, using a conventional Mössbauer spectrometer (WissEl, Starnberg, Germany) operating in the constant acceleration mode with ^57^Co source in Rh matrix. Low-temperature (*T* = 5 K) Mössbauer spectroscopy measurements in a magnetic field parallel to the radiation direction were performed in a cryostat (CRYOGENIC LIMITED). The system allows measurements in the range of 2–300 K and 0–7 T. OLTWINS Mössbauer spectrometer based on (Procházka et al. 2020) was used for the in-field measurements. The specification of the equipment required application of slightly higher concentration (final iron concentration 50 mM). However, preliminary Mössbauer analysis at 80 K showed that such increase of concentration does not cause significant difference in the spectrum.

The spectra were subjected to standard computer-based statistical analysis techniques, involving the MossWinn program (Klencsár 2019) to fit the experimental data through a least-squares minimization procedure. The parameters calculated for the spectral components correspond to hyperfine parameters of Mössbauer nuclei such as isomer shift (*δ*), quadrupole splitting (*Δ*), linewidth (*Г*) and partial resonant absorption areas. For the paramagnetic hyperfine structure model additionally the coefficients of the second order zero field splitting term and the diagonal element of the isotropic hyperfine magnetic interaction tensor *A* were determined. ^57^Fe isomer shifts are given relative to α-iron at room temperature.

### Electron paramagnetic resonance spectroscopy

X-band electron paramagnetic resonance spectroscopy measurements were performed using a Bruker ElexSys E500 X-band spectrometer on the frozen solutions. The EPR measurements were conducted under the following conditions, unless stated otherwise: a modulation frequency of 100 kHz, modulation amplitude of 1 G, microwave power of 2.07 mW and microwave frequency of *f* ≈ 9.3 GHz. The samples with a volume of 400–500 μL were transferred to the EPR quartz tube sample holder and slowly cooled down to 150 K with a mean cooling rate of ca. 30 K/min. For the fast-freezing experiment, the same procedure as for Mössbauer measurements was applied. The sample geometry with respect to the EPR cavity and the spectrometer settings were kept uniform for all the samples, ensure consistency in the signal amplitude and enable meaningful comparisons of EPR signal intensities. A separate spectrometer background reference was measured by using 500 μL of distilled water (frozen at 150 K), and was subsequently subtracted from the measured spectra prior the further analysis.

## Results and discussion

### Iron(III) citrate complexes in aqueous solution

**Fig. 1:**
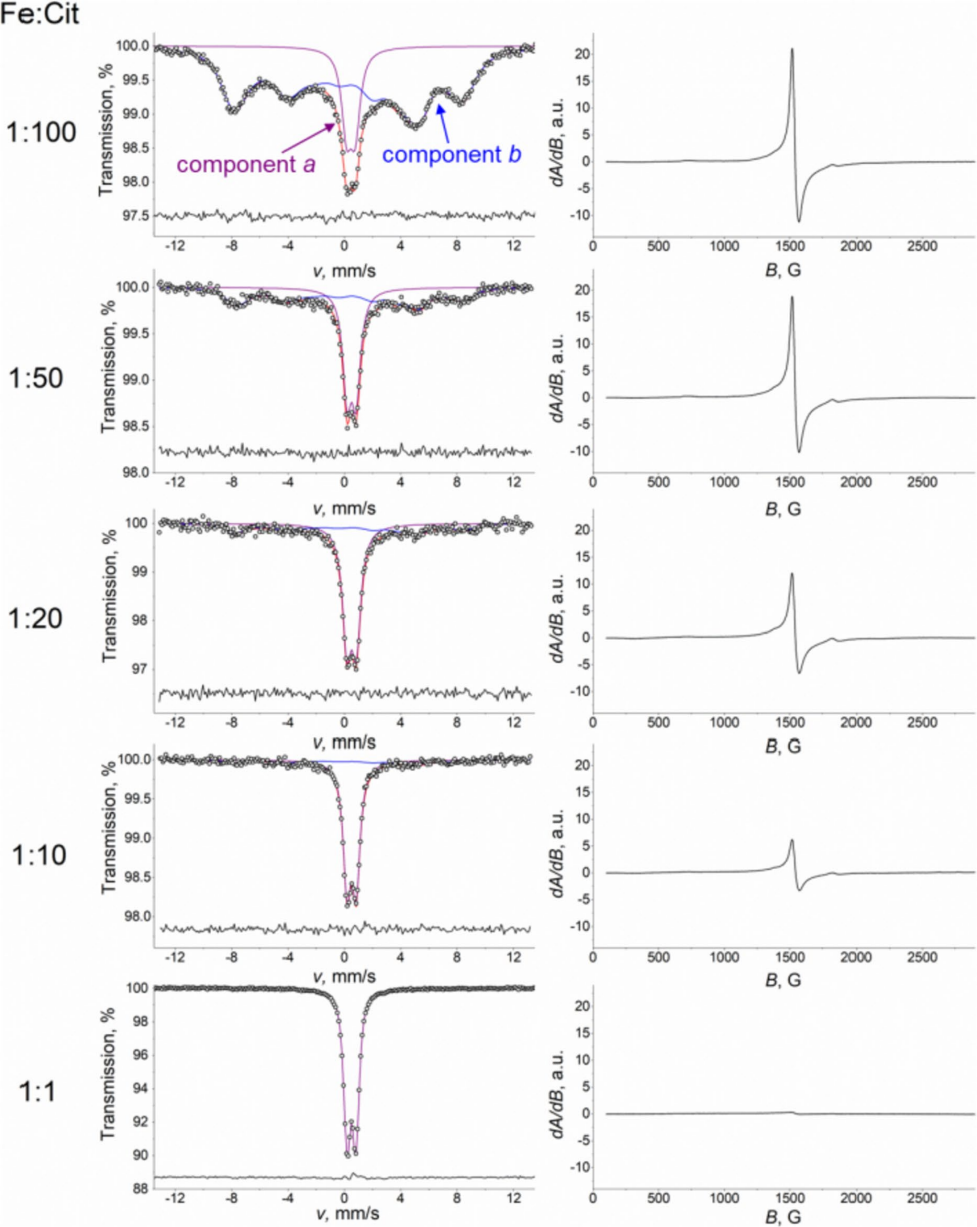
Mössbauer (*T* = 80 K) and EPR (*T* = 150 K) spectra of iron(III) citrate frozen solutions at pH 5.5 at different Fe-to-citrate molar ratios

^57^Fe Mössbauer spectra of rapidly frozen solutions of iron(III) citrate differing in Fe-to-citrate molar ratio (Fe:Cit) measured at 80 K (Fig. 1, left) allowed to distinguish two main constituents: a quadrupole doublet (component *a*) and a peculiar magnetic component fitted using the paramagnetic hyperfine structure (PHS) model (component *b*) (Klencsár 2019).

The appearance of paramagnetic hyperfine structure is to be expected for mononuclear iron(III) species subject to slow electronic relaxation with respect to the Larmor precession time (*τ*_L_) of the ^57^Fe nucleus. Under such circumstances the nuclear magnetic moment can couple strongly to the electronic moment giving rise to a Mössbauer component displaying magnetic splitting. The absence of an orbital magnetic moment of high-spin Fe^3+^ leads to a temperature dependent spin–lattice relaxation rate that is expected to be low enough for the observation of PHS already at *T* = 80 K. On the other hand, spin–spin relaxation rate can also be kept low by applying a suitably low concentration (~ 0.5 M and below) of iron in the solution (Mørup et al. 1976; Vértes and Parak 1972).

Consequently, in line with the iron concentration of 0.01 M applied in the case of the present samples, the analysis of the component *b* reflecting paramagnetic hyperfine structure was performed in the slow electronic relaxation limit; i.e. by assuming that the rate of spin state transitions is low enough to have only negligible effects on the measured spectra. In addition, the fitting was performed in assumption of powder geometry and an effective spin of *S* = 5/2 along with the following spin Hamiltonian:

1 $${\varvec{H}}=D\left[{{\varvec{S}}}_{z}^{2}-\frac{1}{3}S\left(S+1\right)+\uplambda \left({{\varvec{S}}}_{x}^{2}-{{\varvec{S}}}_{y}^{2}\right)\right]+{g}_{{\text{N}}}{\mu }_{{\text{N}}}A{\varvec{S}}{\varvec{I}}+\frac{eQ{V}_{zz}}{4I\left(2I-1\right)}(3{{\varvec{I}}}_{z}^{2}-I\left(I+1\right)+\eta ({{\varvec{I}}}_{x}^{2}-{{\varvec{I}}}_{y}^{2}))$$

where *D* stands for the coefficient of the second order zero field splitting (ZFS) term describing uniaxial distortion of the cubic crystal field, $\lambda =E/D$ denotes the rhombicity parameter (with *E* being the coefficient of the second order ZFS term describing rhombic distortion), ${\mu }_{{\text{N}}}$ is the nuclear magneton, ${g}_{{\text{N}}}$ is the nuclear *g* factor, *I* is the nuclear spin, *A* is the diagonal element of the isotropic hyperfine magnetic interaction tensor, $e$ is the charge of the proton, $Q$ is the nuclear quadrupole moment, ${V}_{zz}$ is the main component of the electric field gradient tensor with the asymmetry parameter *η* (Vértes and Nagy 1990). The quantitative information for the iron molecular species can be obtained from the relative spectral areas of the subspectrum components *a* and *b* (Fig. 1), considered to be approximately proportional to the fraction of iron atoms contributing to the respective molecular species.

As shown in Fig. 1, as the concentration of citric acid decreased, the relative area of the magnetic component *b* decreased gradually and totally disappeared at the Fe-to-citrate ratio 1:1, leaving only the doublet component *a* with isomer shift *δ* = 0.48(1) mm/s and quadrupole splitting *Δ* = 0.63(1) mm/s, which values are close to those observed earlier for trinuclear iron(III) carboxylate complexes in solid samples (Long et al. 1973). The component *a* can be associated with polynuclear iron molecular species where the shorter distance between iron ions leads to increased spin–spin relaxation rates and an associated absence of magnetic splitting in the spectra. On the other hand, the spectrum component *b* displaying magnetic splitting, therefore, can be associated with mononuclear high-spin Fe^3+^ species undergoing slow magnetic relaxation due to low spin–spin relaxation and—at the applied temperature—negligible spin–lattice relaxation rates.

The attribution of the component *b* to monomer units is further corroborated by the X-band EPR spectra measured on the same frozen solutions at *T* = 150 K. The main component of these spectra (Fig. 1, right) is a signal at *g*_eff_ ≈ 4.3 (at *B* ≈ 1540 G) that is typical for mononuclear ferric compounds with a ligand environment symmetry deviating from that of octahedral due to rhombic distortions (Oosterhuis 1974; McGavin and Tennant 1985; Klencsár and Köntös 2018). Polynuclear complexes containing oxygen-bridged ferric ions were shown to be silent in the same region of the EPR spectrum due to spin coupling (Evans et al. 2008; Silva et al. 2009a).

The intensity of the EPR signal associated with the paramagnetic monomer units decreases with increasing Fe-to-citrate ratio as shown in Fig. 1. This clearly indicates that an increase in the Fe-to-citrate ratio leads to a reduction in the concentration of mononuclear iron(III) species in the samples, and thereby corroborates that the magnetic component *b* of the Mössbauer spectra (Fig. 1) indeed originates from mononuclear iron(III) citrate complexes.

For the highest Fe:Cit ratios applied (1:10 and 1:1), in the EPR spectra one can still observe an EPR signal of mononuclear complexes, though compared to the samples with lower Fe:Cit ratios the corresponding signal intensity is strongly reduced, especially in the case of the sample with Fe to citrate 1:1. In contrast, in the Mössbauer spectra of the same samples the magnetic component is hardly detectable anymore. For this, the amplitude of the corresponding absorption signal must be in or below the range of the amplitude of statistical noise in these Mössbauer measurements. In comparison, the EPR signal of polynuclear species, represented by a broad peak centred at around 3.2 kG (*g* ≈ 2.1) with a peak-to-peak width of ca. 1.1 kG as depicted in Figure S0, remains small in intensity even for the highest Fe:Cit = 1:1 ratio applied (Figure S1), suggesting that majority of Fe^3+^ ions remain EPR silent in polynuclear species, presumably due to the prevalence of strong antiferromagnetic Fe^3+^—Fe^3+^ interactions.

The EPR measurements depicted on Fig. 1 were carried out by cooling the liquid samples from room temperature to 150 K in several minutes, i.e. in a relatively slow manner. In contrast, the Mössbauer spectra of Fig. 1 were recorded on frozen samples obtained by dripping droplets of the solutions into small circular depressions of an aluminium block cooled to ~ 80 K via the use of liquid nitrogen. The latter method is thought to be fast enough to preserve the state and relative occurrence of mononuclear and polynuclear iron(III) citrate complexes of the liquid state in the resulting frozen samples (Vértes and Parak 1972; Vértes and Nagy 1990; Müller et al. 2019; Gracheva et al. 2022).

In order to investigate the possible effects of the rate of cooling on the EPR spectra of the frozen solutions, droplet samples (with Fe-to-citrate ratios of 1:1 and 1:100) were frozen in the same way as applied in the case of the Mössbauer measurements and measured at 150 K via EPR spectroscopy. The EPR spectra of the respective slowly cooled and fast frozen samples (Suppl. Figure S2, S3) did not show significant differences regarding the relative signal amplitudes of mononuclear and polynuclear iron complex species, or regarding the spectral shape of the signal associated with the mononuclear units, suggesting that for the investigated complexes at the applied concentrations the rate of freezing does not play a significant role.

**Fig. 2:**
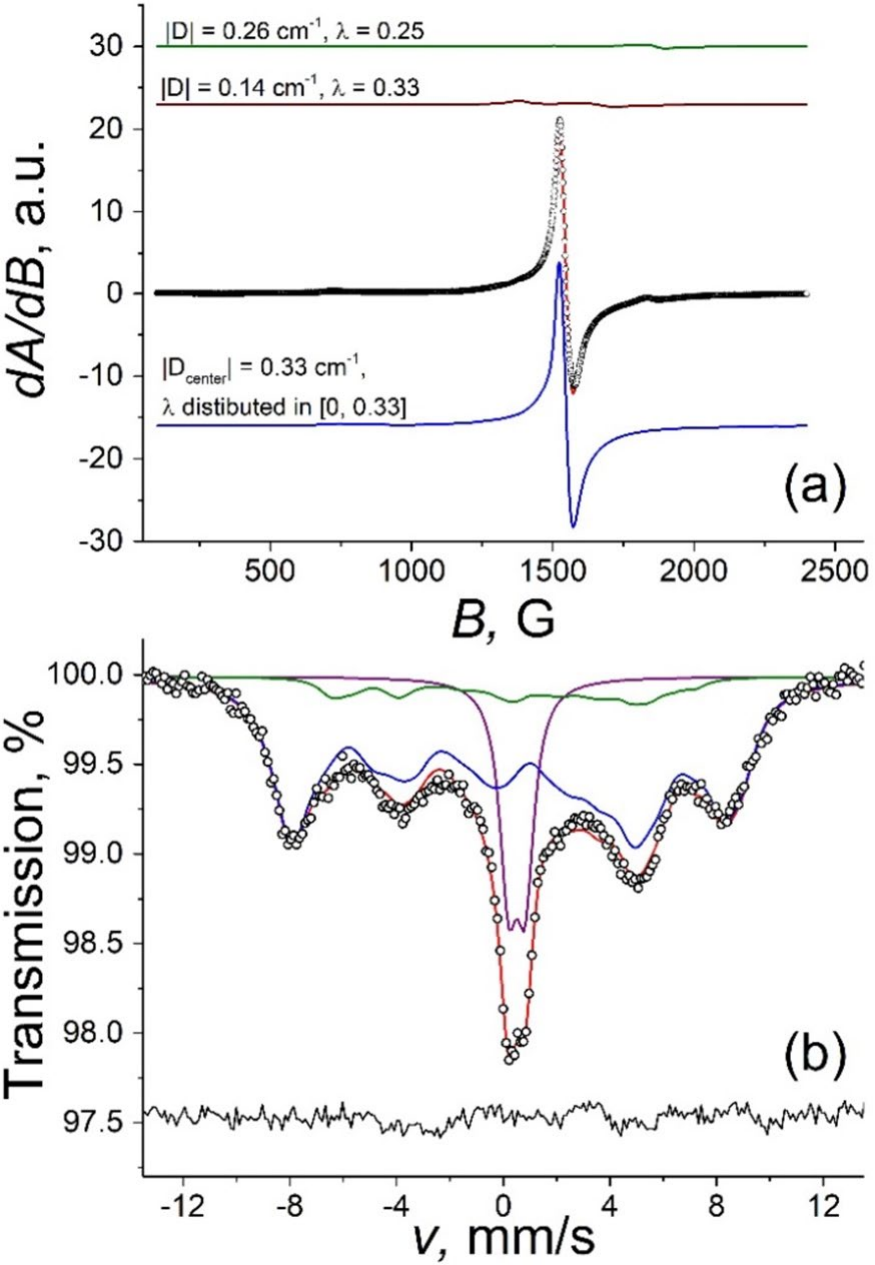
Magnified view of the X-band EPR spectrum (**a**) and Mössbauer spectrum (**b**) of iron(III) citrate frozen solution with Fe:Cit = 1:100 along with several different subspectra possibly contributing to the spectrum due to the presence of mononuclear iron(III) citrate complexes with different ligand environments

Beside the main EPR signal component at *g*_eff_ ≈ 4.3, in the EPR spectra (Fig. 1, 2) one can also observe smaller intensity signals as its shoulders towards lower as well as higher magnetic field values. This refers to the presence of mononuclear iron(III) complexes having different ligand symmetries resulting in different values of the spin Hamiltonian ZFS parameters of *λ* and *D*. In order to illustrate how these may contribute to the resulting spectra, EPR model spectra were calculated as described in ref. (Klencsár and Köntös 2018) for ^56^Fe. Obtained values of *λ* and *D* and corresponding theoretical spectra are depicted in Fig. 2 together with the EPR spectrum of the sample having Fe:Cit = 1:100. The main signal component (marked with blue) was described by applying a distribution of the rhombicity parameter *λ*. The distribution reaches its maximum at the value *λ* = 0.33 for all samples (Supp. Figure S4). The individual distribution data points are also associated with a Gaussian distribution in parameter *D*. This latter distribution has a centre of ca. -0.33 cm^−1^. The distribution of the ligand field parameters refers to the presence of slightly different mononuclear iron(III) species (Klencsár and Köntös 2018). Such high variety of iron(III) ligand environments is presumably realized due the effect of molecules in the second coordination sphere of the complex, which may influence the symmetry of the ligands in the first coordination sphere. The other two components possibly contributing to the spectrum have parameters |*D*| ≈ 0.14 cm^−1^, *λ *≈ 0.33 and |*D*| ≈ 0.26 cm^−1^, *λ *≈ 0.25.

In the respective Mössbauer spectra (Figs. 1, 2) the rather broad absorption peaks of component *b* associated with monomer units in part may also be a consequence of the presence of a multitude of slightly different ligand spheres in iron citrate monomers. At the same time, the width excess of the same absorption peaks may also be contributed to by spin relaxation effects. Therefore, the decomposition of component *b* obtained in the Mössbauer spectrum of the sample with Fe:Cit = 1:100 (Fig. 2) was performed by assuming a variable line width (FWHM) parameter meant to account formally for the above-mentioned broadening effects.

In agreement with the evaluation of the obtained EPR signals, the major area (~ 90%, marked with blue) of the component *b* of the Mössbauer spectrum can be described by a single component using the paramagnetic hyperfine structure model assuming slow electronic relaxation along with *D* ≈ − 0.34 cm^−1^ and a distribution of parameter *λ* (Fig. 2, Suppl. Table S1). The shape of the calculated distribution is consistent with the one observed in the case of EPR evaluation (Suppl. Figs. S4, S5). The second component (marked with green) has much smaller relative area and exhibits parameters of *D* ≈ − 0.18 cm^−1^ and *λ *≈ 0.33. The isomer shifts of both components were close to 0.59(2) mm/s as expected for high-spin iron(III) compound at low temperature. The high value of error and slight disagreement with the data obtained from EPR may be caused by the very large value of line width and overlapping of components.

The analysis of the data obtained from mass spectroscopy and spectrophotometry experiments (Gautier‐Luneau et al. 2005, Evans et al. 2008; Silva et al. 2009a) indicated the potential simultaneous presence of multiple mononuclear species of iron citrate: the partially coordinated FeLH and the fully coordinated FeL_2_, FeL_2_H, FeL_2_H_2_, where the ligand L is defined as the tetrabasic citrate ion C_6_H_4_O_7_^4−^. Results of the fittings of both Mössbauer and EPR spectra showed that the dominant component in all cases exhibited extreme rhombic symmetry (*λ* ≈ 0.33) and may correspond to the fully coordinated complexes. The theoretically calculated speciation plots shown in ref. (Silva et al. 2009a) suggest that complexes FeL_2_, FeL_2_H, FeL_2_H_2_ can be present simultaneously in Fe citrate solution at pH 5.5 with a slight prevalence of FeL_2_H. Since the closest microenvironments of Fe nuclei in all of these complexes are very similar, there is no possible way to resolve the contribution of different individual species and the distribution of the ZFS parameters was observed. The dominance of the fully coordinated monomers is also consistent with the indifference of the EPR spectrum shape to the rate of freezing. Namely, partially coordinated complex FeLH, where the first coordination sphere of iron needs to be completed via the involvement of water molecules, would be more affected by a change in the freezing rate due to the ice formation. The component marked with green in the EPR spectrum and exhibiting reduced rhombicity may be attributed to the FeLH complex, which is suggested by Silva to be present as a minor phase. It is remarkable that while the mentioned theoretical plot does not exhibit dramatic difference between different monomeric species at different Fe-to-citrate ratios, the calculation performed in ref. (Evans et al. 2008) suggests that there should be a much more significant contribution from the monoligand complex that decreases with citric acid concentration. However, the latter calculation does not consider polynuclear species.

**Fig. 3:**
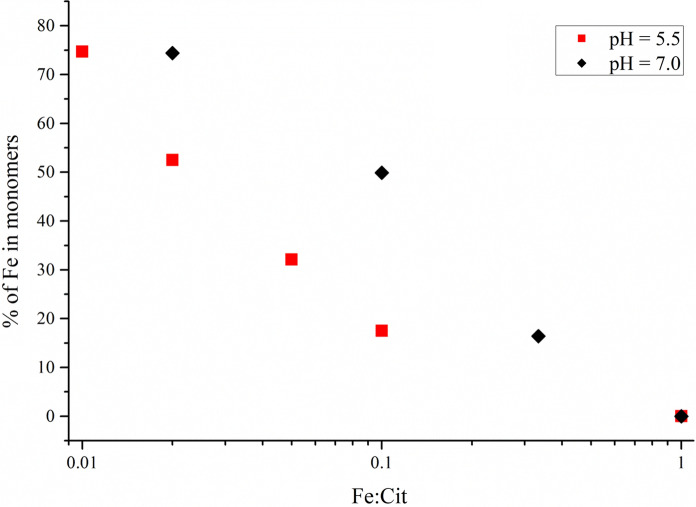
Relative area of the component associated with mononuclear complexes at different Fe-to-citrate molar ratios and pH values. Error bars are smaller than the symbols

Considering the significant biological relevance of the studied complexes, additional samples were prepared at a neutral pH of 7.0 with Fe:Cit = 1:1, 1:3, 1:10 and 1:50. The Mössbauer spectra of the new solutions (Suppl. Fig. S6) exhibited similar components. However, the relative areas of these components varied compared to those observed at the same iron concentrations for pH 5.5. (Fig. 3). A detailed comparison of the obtained dependence with the theoretical speciation plots (Silva et al. 2009a) shows a slight disagreement between the observed and suggested ratios of polynuclear and mononuclear complexes. Although the general trend of increase in the relative amount of the monomers with the increase of pH or/and increase of citrate concentration is the same for theory and our observation, the experimental results in this study show much more dramatic change in the ratio of Fe incorporated in monomers than predicted earlier. While Silva et al. (Silva et al. 2009a) suggested more than 70% of Fe to be incorporated into polynuclear species at pH 5.5 regardless of Fe-to-citrate ratio (calculation were performed for 1:10 and 1:100), we did not observe the dominance of oligomers at high citrate excess. This disagreement may be caused by higher concentration values applied in this work, which was a technical requirement.

The dependence obtained (Fig. 3) showed that for all ratios except 1:1 the relative amount of iron coordinated in monomers was higher at pH 7.0 than at pH 5.5. This observation may be explained by the deprotonation of the citric acid. As the p*K*_a_ values for the three carboxylic acid functional groups are p*K*_a1_ = 3.13, p*K*_a2_ = 4.76, p*K*_a3_ = 6.40 and p*K*_a4_ = 14.4 (Silva et al. 2009a, b), the raise of pH from 5.5 to 7.0 leads to a higher coordination ability of the citrate ligands which facilitates the formation of monomers. However, it is possible only when citrate is available in high excess.

The presence of a single doublet and strongly diminished EPR signal at Fe-to-citrate molar ratio 1:1 was observed in a broad pH range (from 3.0 to 7.5) and iron concentration range (from 10 to 150 mM), suggesting that at Fe:Cit = 1:1, the strong need for coordination saturation of Fe^3+^ by citrate requires the formation of polynuclear structures, and the effect of pH and iron concentration in the mentioned range is negligible. Taking a closer look on the observed Mössbauer doublet, one can notice that its linewidth is quite large *Г* = 0.55(3) mm/s. Earlier authors (Epstein et al. 1970) suggested that the broadness of the lines is a consequence of iron being present in a variety of slightly different environments, which in turn is also caused by the polynuclear structure of the studied complexes.

Among the suggested complexes (Pierre and Gautier-Luneau 2000; Ganz et al. 2019), polynuclear entities are most expected to be presented as dimers or/and trimers. However, a dimer with oxygen bridged Fe ions, as suggested in (Shweky et al. 1994), are known to be observed in Mössbauer spectrum as a doublet with sharp lines and large quadrupole splitting (Vértes and Parak 1972; Sharma et al. 2005). Therefore, trimers are suggested to be the dominant species in the described case. To clarify the properties of the polynuclear species, the samples of Fe-to-citrate ratio 1:1 and 1:10 were measured additionally at 5 K with and without application of external magnetic field.

**Fig. 4:**
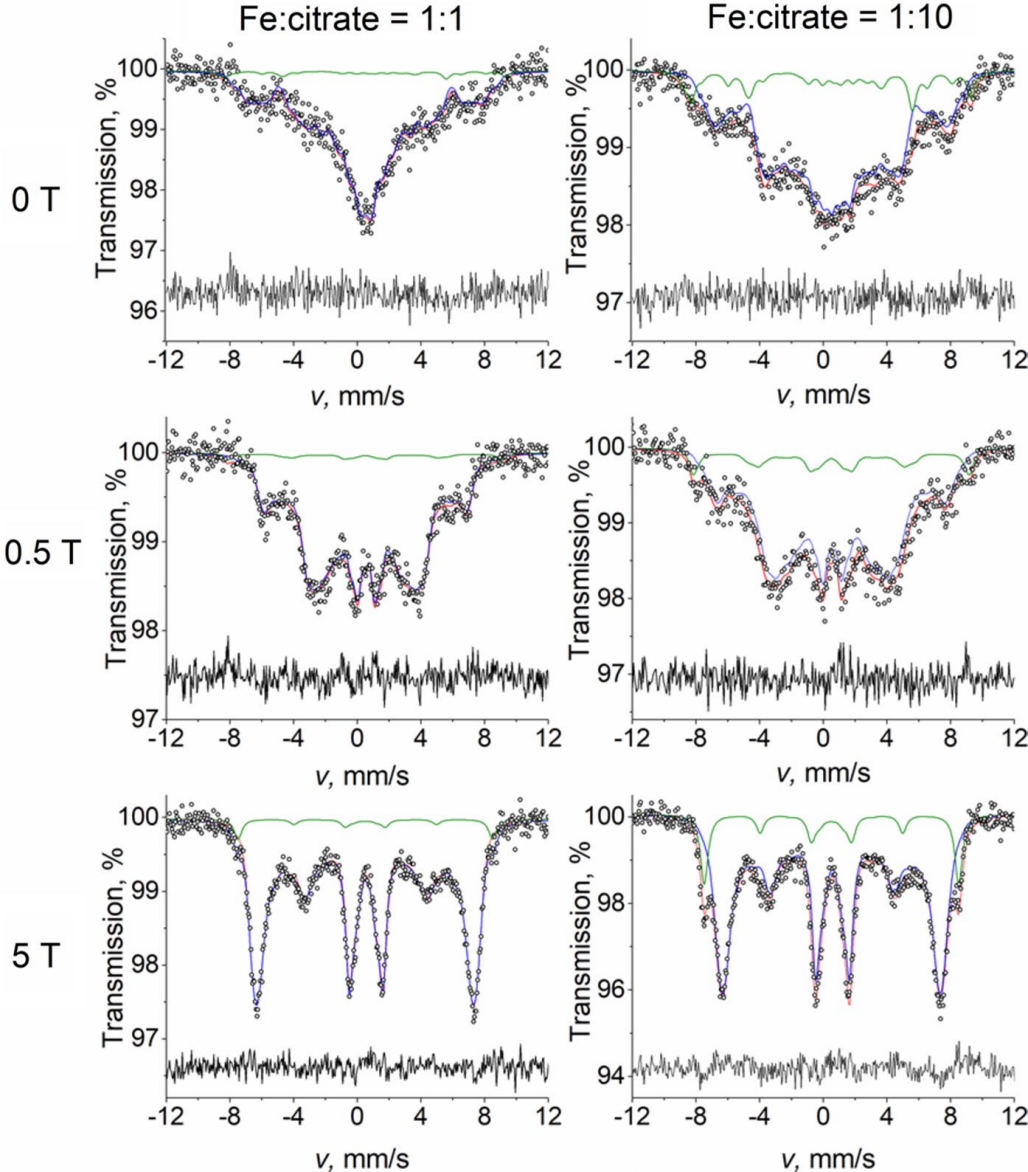
Mössbauer spectra of iron(III) citrate frozen solutions at different Fe-to-citrate molar ratios with and without external magnetic field. Spectra were measured at *T* = 5 K

Application of the external magnetic field causes the energy splitting of ionic spin levels to depend on the angle between the applied field and the ligand field axis. Since the investigated samples were frozen solutions, ensuring the random orientation of the Fe entities, an application of external magnetic field may result in an increase of the spin–spin relaxation time, making the spectrum lines sharper, as was observed (Mørup et al. 1978). The cross-relaxation theory described in (Vértes and Nagy 1990; Mørup et al. 1978) suggests that increasing the applied magnetic field leads to the decrease of relaxation frequency. Indeed, in our case we have observed that while application of 0.5 T just causes the satellite peaks of the broad magnetic component to be more defined, the increase of magnetic field value up to 5 T allows to identify different lines of the sextet separately, suggesting that the relaxation time is high enough (Fig. 4). The fitting was performed with a model consisting of a PHS component for monomeric species (marked with green in Fig. 4) and a magnetic sextet with a probability distribution of magnetic field for polynuclear species (marked with blue). In the in-field spectrum of the solution with Fe-to-citrate ratio 1:10, a very clear appearance of the PHS component can be observed. Its relative area is around 15%, which is in good agreement with the relative amount of Fe coordinated to monomeric species at Fe:Cit = 1:10 observed earlier in the Mössbauer measurements at 80 K. Although the previous Mössbauer spectrum of the equimolar solutions did not show the presence of monomers because its relative area was lower than the statistical noise, the parallel evaluation of in-field spectra of the samples Fe:Cit = 1:1 and 1:10 allowed to determine that the monomers are present in the equimolar solution with the relative abundance of approximately 4%. The PHS component was found to be insensitive to the variation of ZFS parameters *λ* and *D*, therefore, they were fixed based on the EPR results. However, the in-field measurements were very useful for determining the exact value of the diagonal element of the isotropic hyperfine tensor* A* = -21.8(1) T, which was used for a better evaluation of the 80 K spectrum.

The change of the magnetic sextet (blue in Fig. 4) by applying 5 T external magnetic field resembles the one observed for the spectrum of the trimeric compounds of chloroacetate, formate, benzoate and acetate in the work of Rumbold (Rumbold and Wilson 1973) and of Takano (Takano 1972). It was observed that the external magnetic field of even 5 T did not lead to vanishing of the second and fifth absorption lines of the subspectrum associated with the polynuclear compound (Fig. 4). This phenomenon may refer to a case when the magnetic moment of one of the components of the antiferro- or ferrimagnetic system is not perfectly parallel or antiparallel to the γ-ray propagation direction from the Mössbauer source. Such behavior can be indeed expected from a trimeric complex. As reported by (Bartolomé et al. 2009), the increase of the external magnetic field to 6.5 T led to only minor changes in the intensities of the absorption lines for polynuclear molecular clusters containing coordinated Fe and Dy ions, showing strong resistance to reorientation of the local magnetic moments enforced by the applied field.

### Iron(III) citrate and EDTA complexes in organic solvents

**Fig. 5:**
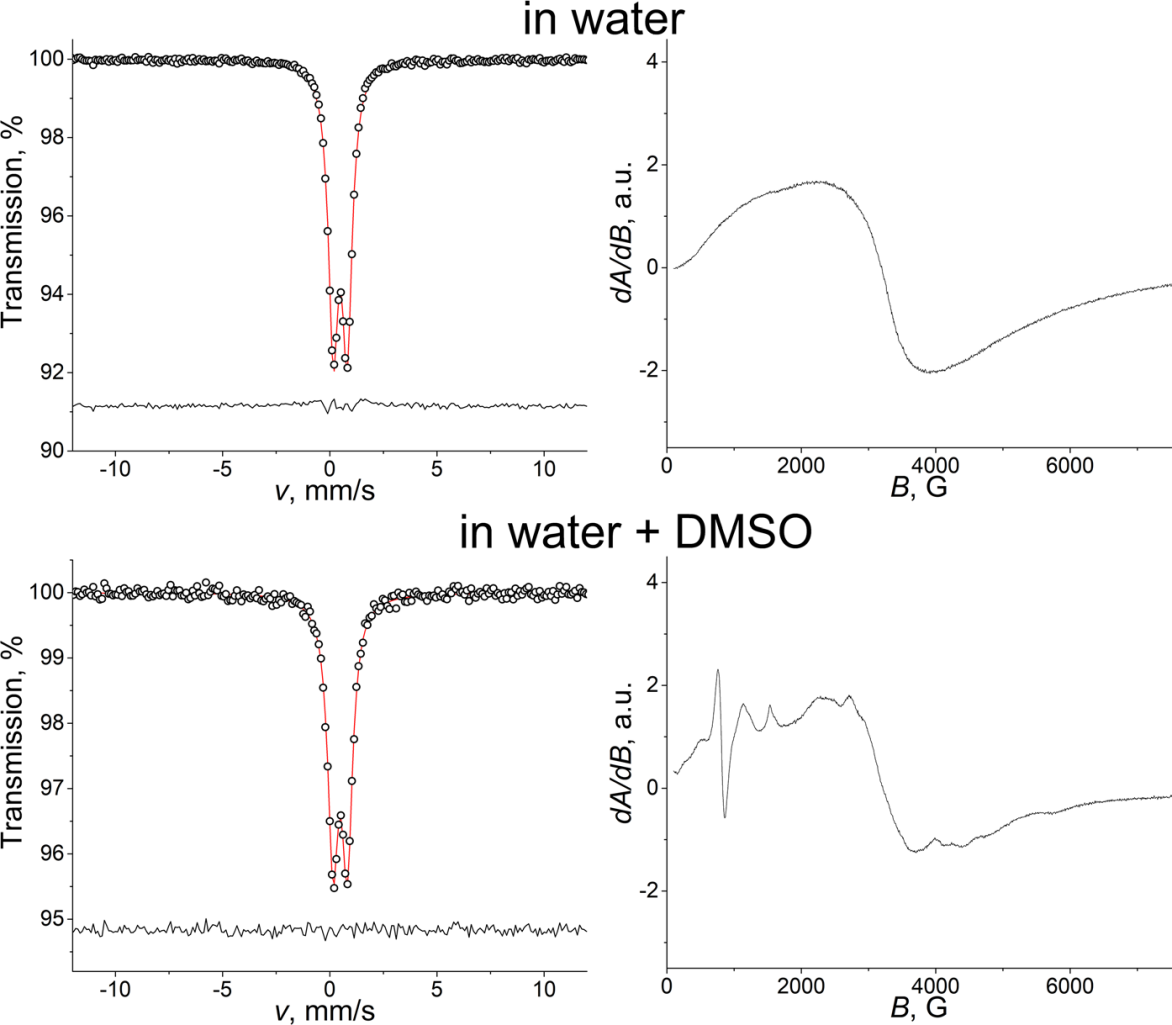
Mössbauer and EPR spectra of iron(III) citrate frozen solutions in the presence of only water and water + DMSO mixture. On the EPR spectrum of the latter characteristic peak maxima are marked with their apparent *g* factor values. The displayed EPR spectra were measured with modulation amplitude of 2 G, and microwave power of 20.7 mW

It was shown earlier that five-fold increase of Fe concentration (up to 50 mM) did not affect the speciation in the case of the equimolar Fe citrate solution, and the two-fold dilution with water also did not lead to any changes in the spectra: the Mössbauer spectrum (Fig. 5) exhibited the presence of only the same slightly broadened doublet, while the corresponding EPR spectrum showed no presence of any well-defined peak in the *g*_eff_ ≈ 4.3 region of paramagnetic Fe species, suggesting that most of the Fe is still incorporated into polynuclear species only. The similar two-fold diluting experiment (final Fe concentration 5 mM) was conducted with Fe citrate and organic solvents (dimethyl sulfoxide (DMSO) and acetonitrile) that are frequently used in biological experiments and showed possible coordination ability to Fe^3+^ (Lázár et al. 2015). However, it was observed that the addition of neither DMSO nor acetonitrile affected the Mössbauer spectrum of Fe citrate (Fig. 5, Suppl. Fig. S7). Disregarding the solvent applied, the spectrum appeared as a doublet with parameters similar to those expected for Fe citrate with trimeric structure. Although DMSO is known to be able to form a trinuclear iron complex with Fe_3_O core, the quadrupole splitting observed for such complex *Δ* = 0.9 mm/s (Wrobleski and Brown 1979) was higher than in our case. Since no broadening of the doublet was detected after addition of DMSO, it may be suggested that the solvent does not affect the polymeric Fe citrate species.

The solutions of Fe citrate in water + DMSO and water + acetonitrile were additionally investigated by means of EPR. Spectra of Fe citrate in aqueous solution and in water + acetonitrile mixture were identical and did not exhibit a presence of the characteristic peak in the *g*_eff_ ≈ 4.3 range of paramagnetic compounds (Suppl. Fig. S7). On the other hand, the sample containing DMSO showed significant difference compared to the other samples (Fig. 5). The peculiar peak pattern appeared at around 1500 G and below did not exhibit any horizontal shift with the increase of temperature to 175 K, suggesting that this peak pattern is indeed associated with paramagnetic species. It can be resolved in a composition of several components; the dominant one has *g*_eff_ ≈ 8.8 and cannot be accounted for simply by the second order ZFS tensor parameters *D* and *λ* = *E/D*. A possible reason for this is the omission of the 4th order ZFS Hamiltonian terms (Vértes and Nagy 1990; Oosterhuis 1974), which would allow for a higher variety of ligand fields and corresponding EPR signal patterns. However, the intensity of the EPR peak pattern appeared due to DMSO is much smaller than that in the spectrum of Fe citrate with the excess of citric acid applied (Fig. 1, Suppl. Fig. S8), suggesting that even after addition of a significant amount of DMSO to Fe citrate (final Fe to DMSO ratio 1:700) the majority of Fe is still incorporated into polynuclear species and the amount of monomers is very small compared to the case of Fe:Cit = 1:100.

**Fig. 6:**
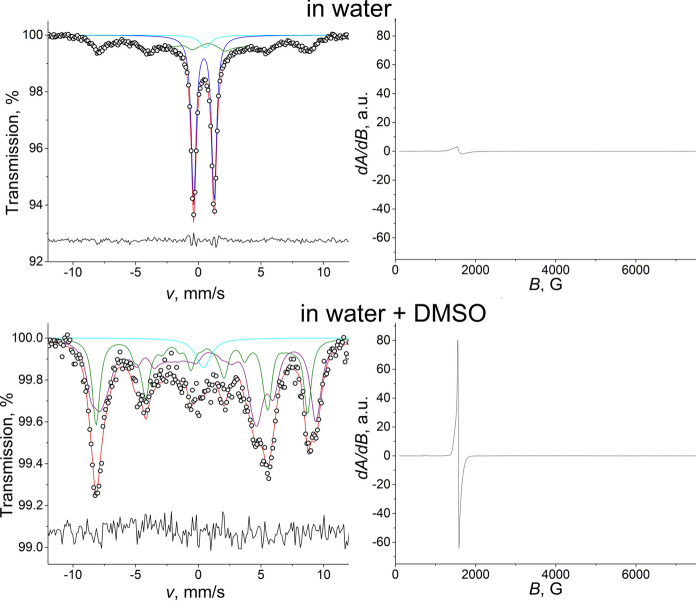
Mössbauer and EPR spectra of iron(III) EDTA frozen solutions in the presence of only water and water + DMSO mixture

To compare the stability of the polynuclear Fe citrate complex, a similar experiment was performed with another ligand known to form beside the monomeric compounds also polynuclear species. The spectrum of Fe-EDTA aqueous solution (pH 5.5, Fe concentration 10 mM, Fe to EDTA ratio was 1:1) showed the presence of a sharp doublet, which can be attributed to the well-known dimeric *µ*-O(Fe^III^EDTA)_2_ complex (Sharma et al. 2005) (Fig. 6). In addition, the spectrum displays also a broad magnetic component of complicated structure appearing due to paramagnetic spin relaxation and corresponding to Fe incorporated in monomeric species (Homonnay et al. 2008). The dilution of the Fe-EDTA aqueous solution to doubled volume with water did not affect the shape of the spectrum suggesting that water does not affect the speciation of Fe at this range of concentrations. On the other hand, the addition of DMSO to the initial Fe-EDTA aqueous solution was found to lead to significant changes. The gradual addition of DMSO (Suppl. Fig. S9) led to a decrease of the relative area of the doublet and the prevalence of the broad magnetic component. The total disappearance of the doublet observed at water-to-DMSO ratio 1:1 (Fe-to-DMSO ratio 1:700) suggests that at this ratio all Fe in the sample is incorporated into mononuclear species. The fitting of the Mössbauer spectrum was performed by using a model consisting of 2 PHS components. Since pH of the solution remained almost constant during all the preparation period, it can be assumed that the addition of DMSO (Suppl. Fig. S9) led to the break of *µ*-oxo bridge in the dimeric structure and coordination of all Fe present in the sample corresponds to monomers. This observation suggests that while Fe citrate trimers preserve their polynuclear structure even in the presence of the organic solvents, Fe-EDTA dimers can be broken by addition of DMSO leading to the formation of monomers as the dominant and single phase. The results of the EPR measurements of the samples confirm the suggestion. Since dimers as antiferromagnetic species do not contribute to the present EPR spectra, a large fraction of iron remains hidden and the EPR signal of the Fe-EDTA aqueous solution originates exclusively from monomers, therefore, remaining small. In contrast, the EPR signal of Fe-EDTA in a mixture of water and DMSO (Fe-to-DMSO ratio 1:700) has much larger intensity, as all Fe is incorporated into paramagnetic mononuclear species and can be observed in the spectrum. It is remarkable to point out that although DMSO is known to form stable mononuclear complexes with iron and iron chloride, such as (Fe(DMSO)_5_Cl)^2+^ and (Fe(DMSO)_6_)^2+^ (Lázár et al. 2015; Gusakovskaya et al. 1983; Solano-Peralta et al. 2009), the EPR spectra of the newly formed complexes in the case of Fe citrate and Fe-EDTA are different suggesting that the initial ligands still play role in the coordination. The addition of other solvent such as acetonitrile to Fe-EDTA solution did not affect the shape of the spectrum and, therefore, was not found to influence the structure and nuclearity of Fe-EDTA complexes.

## Conclusion

The analysis of iron complexation using Mössbauer and electron paramagnetic resonance spectroscopies revealed the coexistence of both polynuclear and mononuclear species at physiological pH. These results provided valuable new insights into the quantitative and qualitative aspects of iron speciation in the presence of citrate excess or organic solvents, which holds significant importance for enhancing our understanding of iron citrate chemistry in biological systems.

Our analysis demonstrated the correlation between the citric acid concentration and the fraction of iron coordinated into mononuclear units. Concentration of the monomer was negligible at high iron to citrate ratios but increased gradually when the ratio is lowered and tended to dominate at iron to citrate ratio of 1:50.

In the case of citrate excess, the comparative evaluation of both EPR and Mössbauer spectra exhibited the presence of several mononuclear species differing in the ligand coordination mode. The dominant components were found to be the fully coordinated monoiron dicitrate complexes. The relative amount of the mononuclear complexes was higher at neutral pH than at slightly acidic conditions which could be explained by a higher coordination ability of citrate due to the deprotonation of the citric acid.

In the case of equimolar iron to citrate ratio, the saturation of the coordination sphere of iron by citrate required the formation of polynuclear structures. Comparing our present results with literature data, polynuclear entities were most probably present as trimers while no oxygen bridged dimeric compound could be found. The effect of pH and iron concentration was shown to be negligible on the polymeric structure which was preserved even after addition of organic solvents.

### Supplementary Information

Below is the link to the electronic supplementary material.

Supplementary file1 (DOCX 1688 kb)

## Data Availability

All datasets generated for this study are included in the article/ supplementary material (as depicted on Figures of the Mössbauer and EPR spectra); further inquiries on row data can be directed to the corresponding author.
