# Supplementary material for: Revealing the nuclearity of iron citrate complexes at biologically relevant conditions
Source: Biometals. 2023 Dec 18;37(2):461–75. doi: 10.1007/s10534-023-00562-1 (PMC11006783; doi:10.1007/s10534-023-00562-1)
Supplement: Supplementary file 1 — Supplementary file1 (DOCX 1688 kb) [file 10534_2023_562_MOESM1_ESM.docx]

**Supplement**

**Revealing the nuclearity of iron citrate complexes at biologically relevant conditions**

Maria Gracheva^1,2^, Zoltán Klencsár^2^, Zoltán Homonnay^1^, Ádám Solti^3^, László Péter^4^, Libor Machala^5^, Petr Novak^5^, Krisztina Kovács^1*^

*^1^Department of Analytical Chemistry, Institute of Chemistry, Eötvös Loránd University Pázmány P. s. 1/A, 1117 Budapest, Pázmány Péter s. 1/A, Hungary*

*^2^ Nuclear Analysis and Radiography Department, Centre for Energy Research, Konkoly-Thege Miklós út. 29-33, 1121, Budapest, Hungary*

*^3^Department of Plant Physiology and Molecular Plant Biology, Institute of Biology, ELTE Eötvös Loránd University, 1117 Budapest, Pázmány Péter s. 1/C, Hungary*

*^4^ Department of Complex Fluids, Institute for Solid State Physics and Optics, Wigner Research Centre for Physics, Konkoly-Thege Miklós út. 29-33, 1121 Budapest, Hungary*

*^5^Department of Experimental Physics, Faculty of Science, Palacký University Olomouc, 17. listopadu 1192/12, 771 46 Olomouc, Czech Republic*

**Corresponding author:* [*krisztina.kovacs@ttk.elte.hu*](mailto:krisztina.kovacs@ttk.elte.hu)


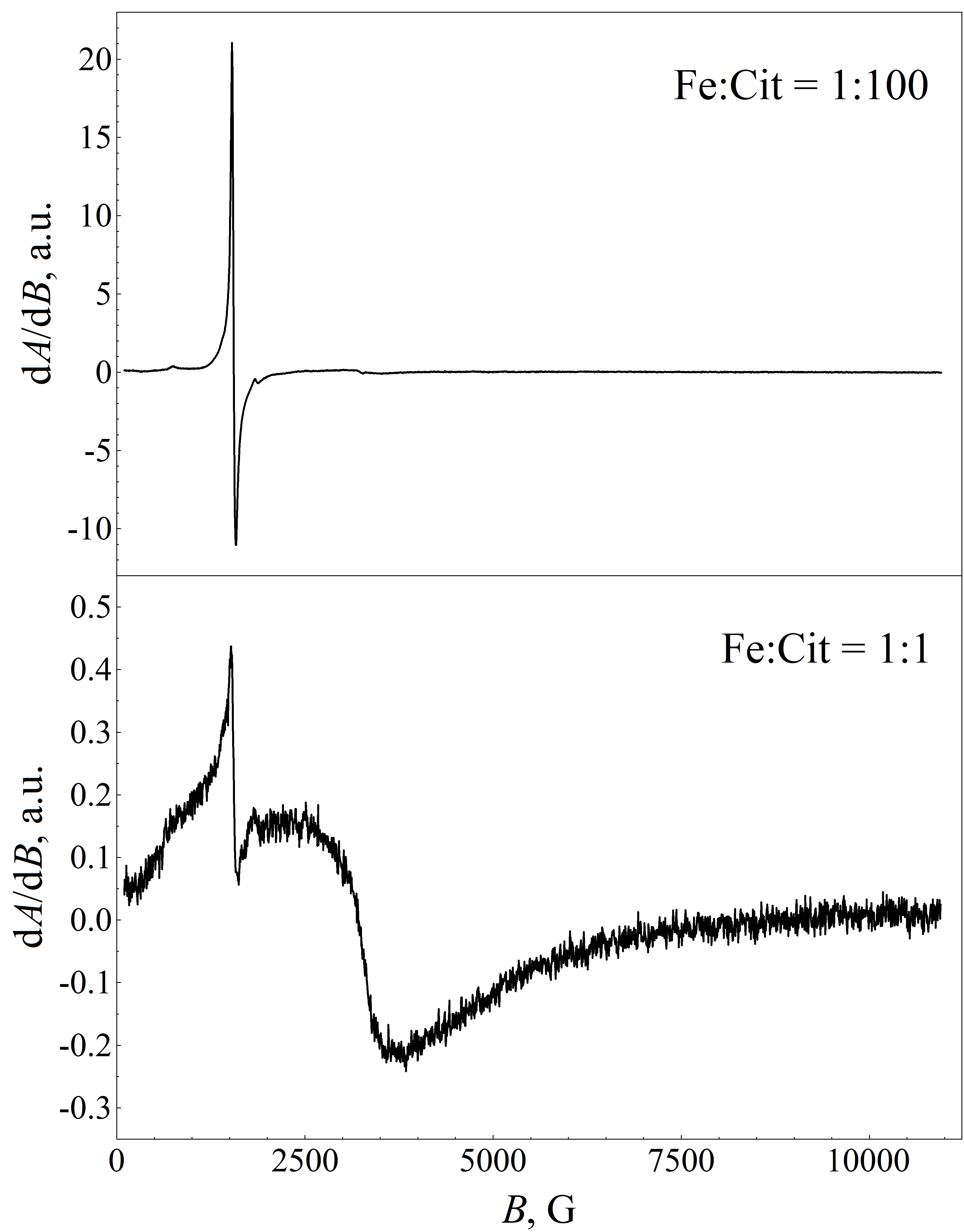


Figure S1. Wide range EPR spectra of ^57^Fe(III) citrate frozen solutions with different Fe:citrate molar ratios at pH = 5.5. Note that a 100 fold increase in the Fe concentration leads to the intensity of the signal of monomers (at around 1500 G) being reduced by a factor of ca. 50. Still, the broad EPR peak (centered at around 3.2 kG) that may be associated with polynuclear species remains low in intensity. This suggests that majority of Fe^3+^ ions remain EPR silent in polynuclear species, presumably due to the prevalence of strong antiferromagnetic Fe^3+^ - Fe^3+^ interactions. The conditions of EPR measurements involved a modulation frequency of 100 kHz, modulation amplitude of 1 G, microwave power of 2.07 mW and microwave frequency of *f* ≈ 9.3 GHz.


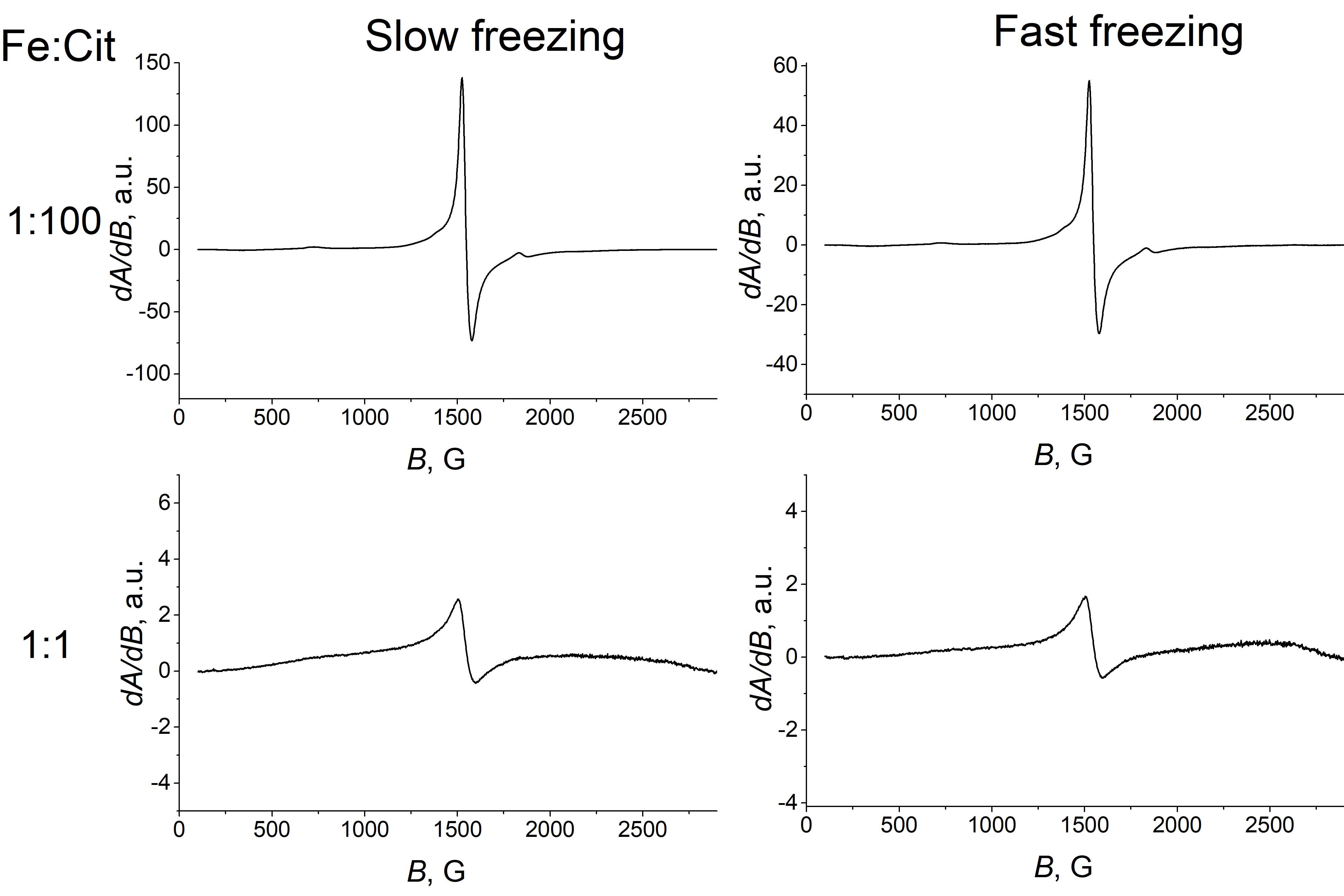


Figure S2. EPR spectra of the frozen solutions with Fe to citrate ratios of 1:1 and 1:100 prepared using methods of slow and fast freezing.


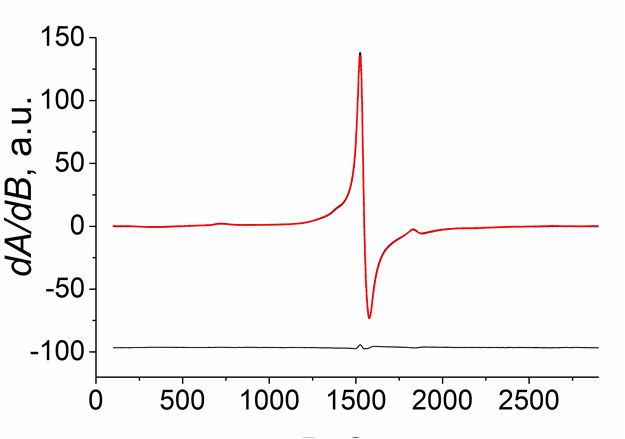


Figure S3. EPR spectrum of solution with Fe to citrate ratio 1:100 prepared by method of slow freezing (black) fitted with the spectrum of solution with the same Fe to citrate ratio prepared by method of fast freezing (red) by multiplying the latter with the appropriate factor for which the two curves provide the least sum of squares. After scaling, they are near identical in the shown range and the differences at around *g* = 2 are also not considerable. The black line at the bottom indicates the difference.


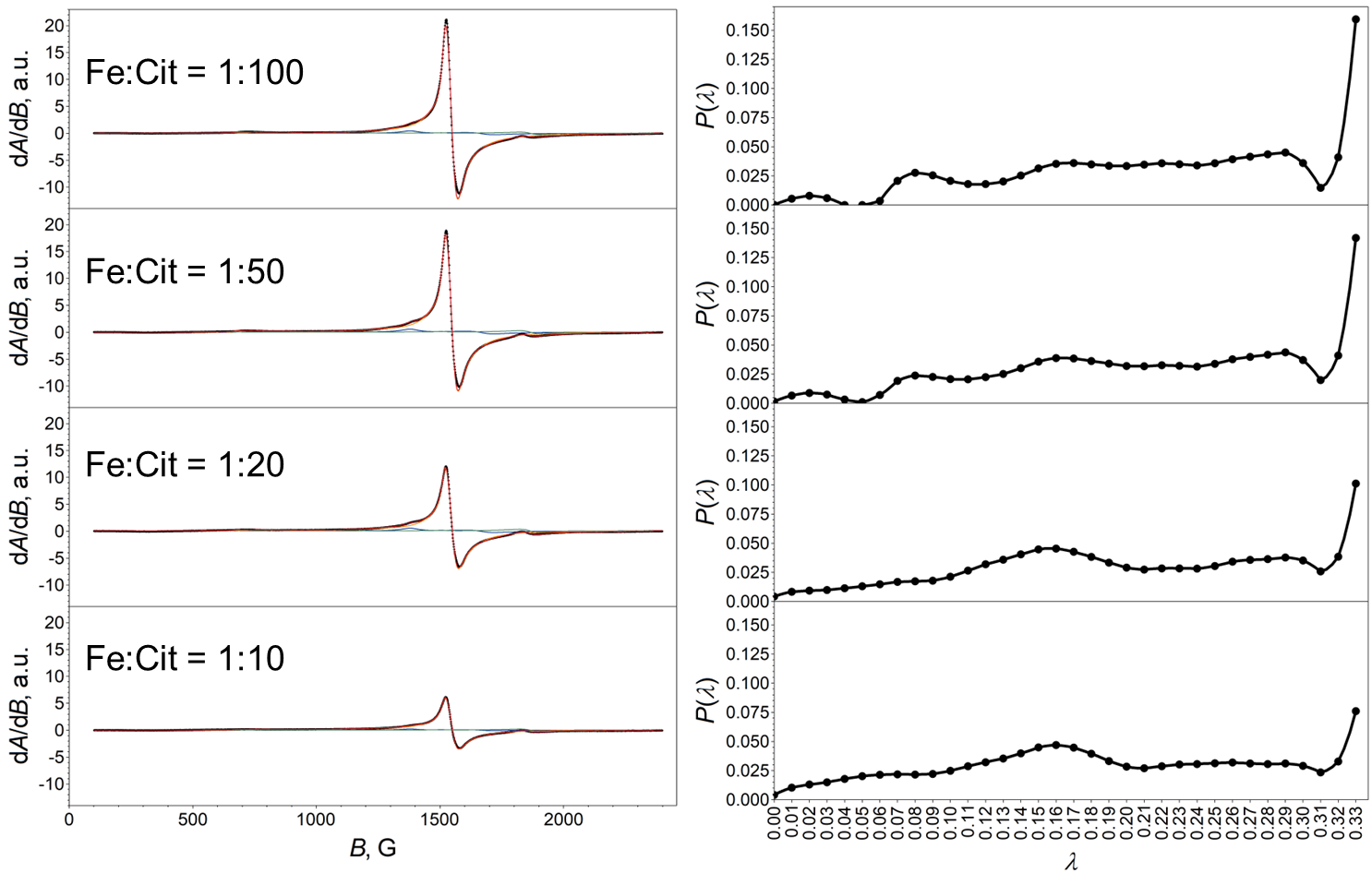


Figure S4. Decomposition of the low-field range signal of the EPR spectrum of the iron(III) citrate frozen solution sample at different Fe to citrate ratios and the corresponding distribution of parameter *λ* for the main component.

|  | Major component (blue) | Minor component (green) |
| --- | --- | --- |
| Isomer shift, mm/s | 0.59(2) | 0.59(fixed) |
| Vzz (EFG), 10^21 V/m^2^ | 6.9(4) | -5.1(9) |
| ETA | 0.54(6) | 1.0(fixed) |
| D, 1/cm | -0.34(1) | -0.18(9) |
| E/D | Distribution | 0.33(3) |
| Axx = Ayy = Azz, T | -21.4(5) | -18(1) |
| Line width, mm/s | 1.41(4) | 1.1(2) |

Table S1. Mössbauer parameters of the PHS components obtained from fitting of the spectrum of iron(III) citrate frozen solution at Fe to citrate ratios of 1:100, at pH = 5.5 measured at *T* = 80 K


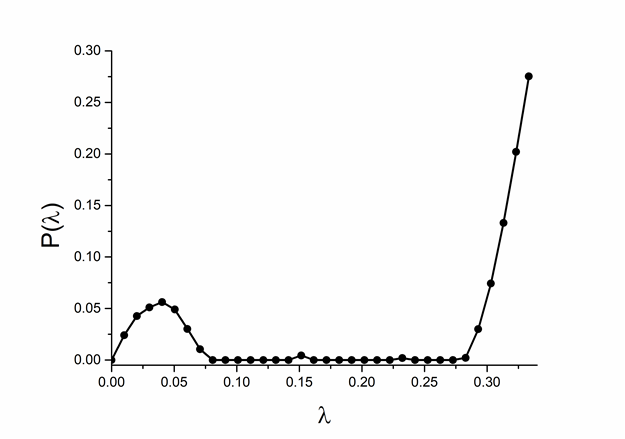


Figure S5. The distribution of the parameter *λ* for the main PHS component of the Mössbauer spectrum of iron(III) citrate frozen solution at Fe to citrate ratios of 1:100, at pH = 5.5 measured at *T* = 80 K


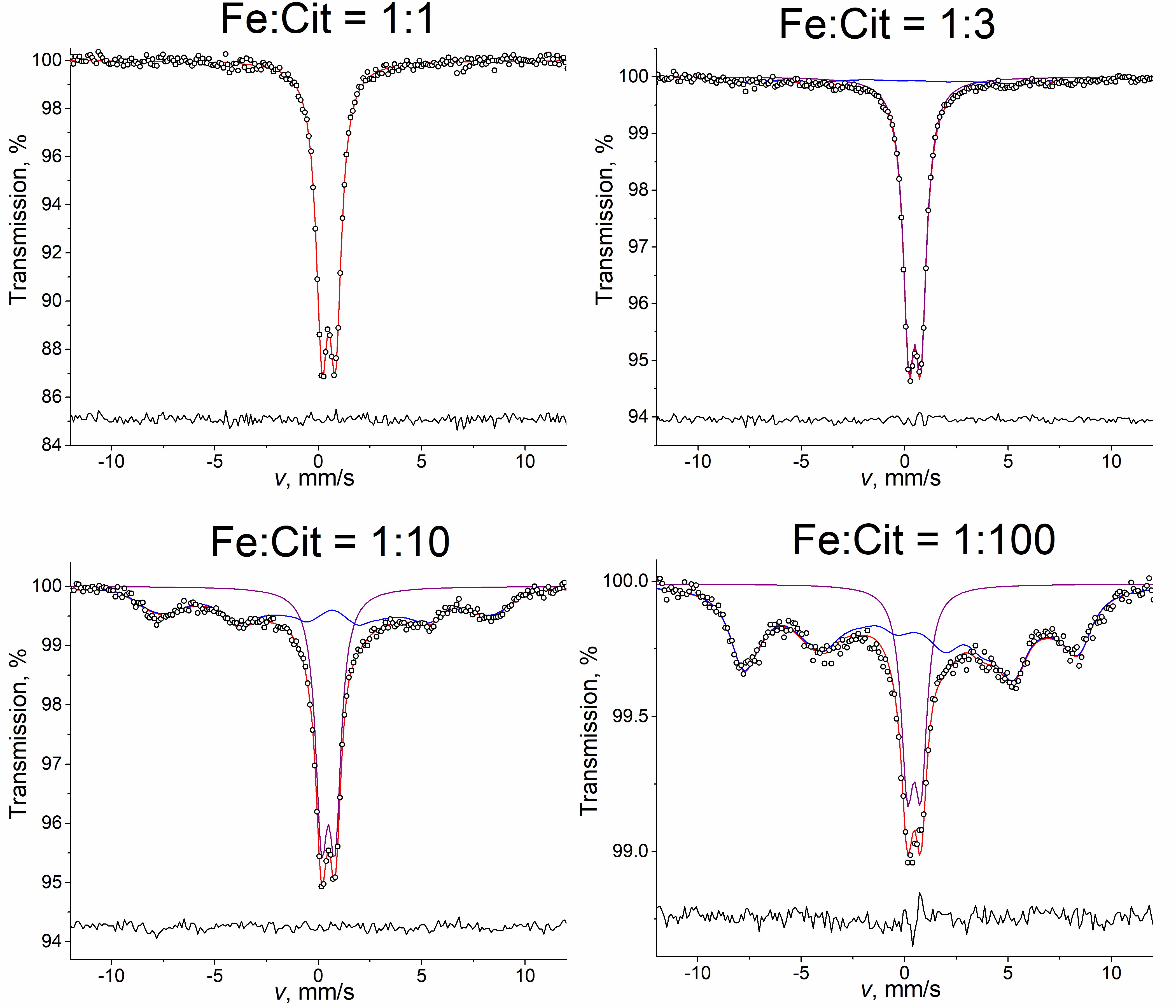


Figure S6. Mössbauer spectra of iron(III) citrate frozen solutions at different Fe to citrate ratios at pH = 7.0


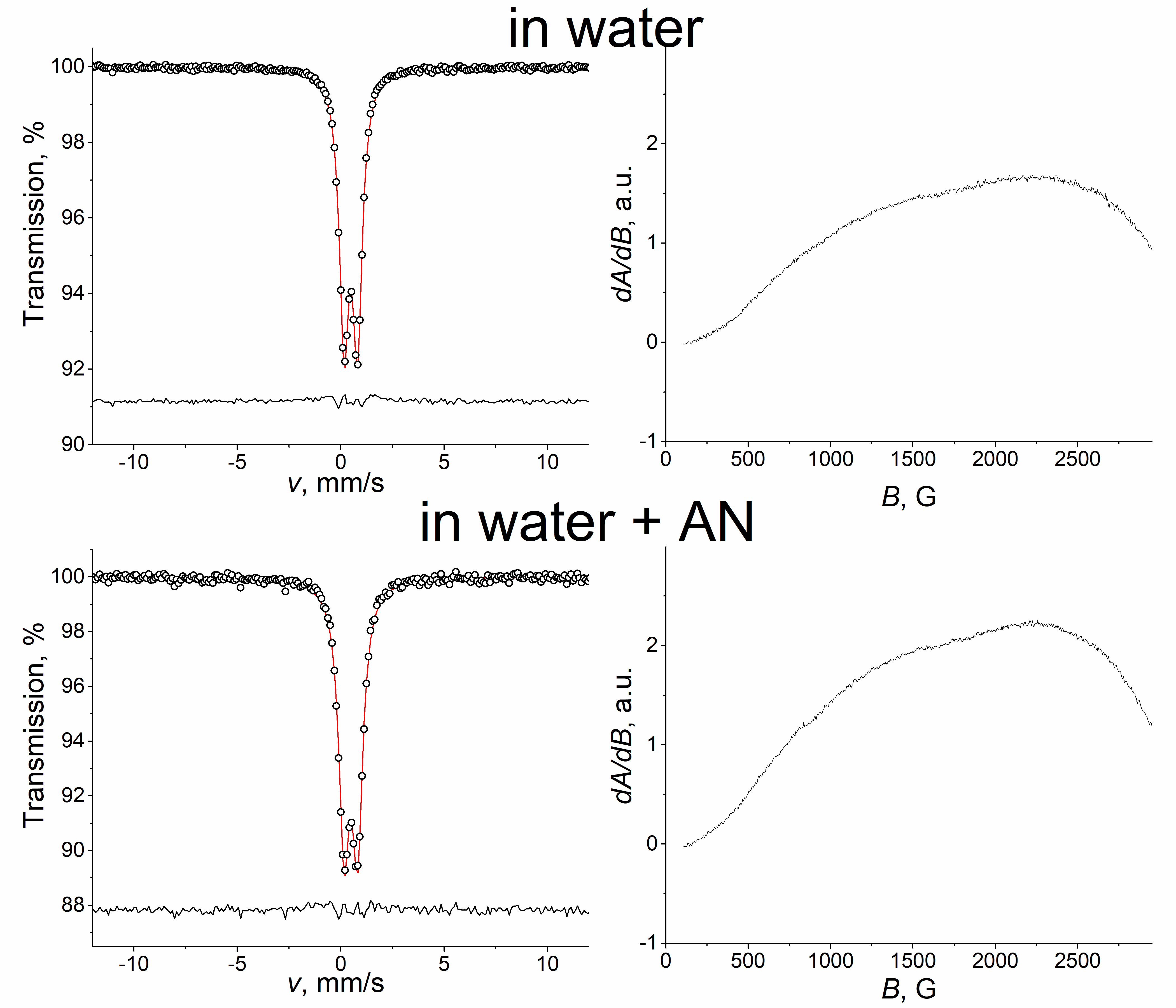


Figure S7. Mössbauer spectra of iron(III) citrate in the presence of only water and water + acetonitrile (AN) mixture. The displayed EPR spectra were measured with modulation amplitude of 2 G, and microwave power of 20.7 mW.


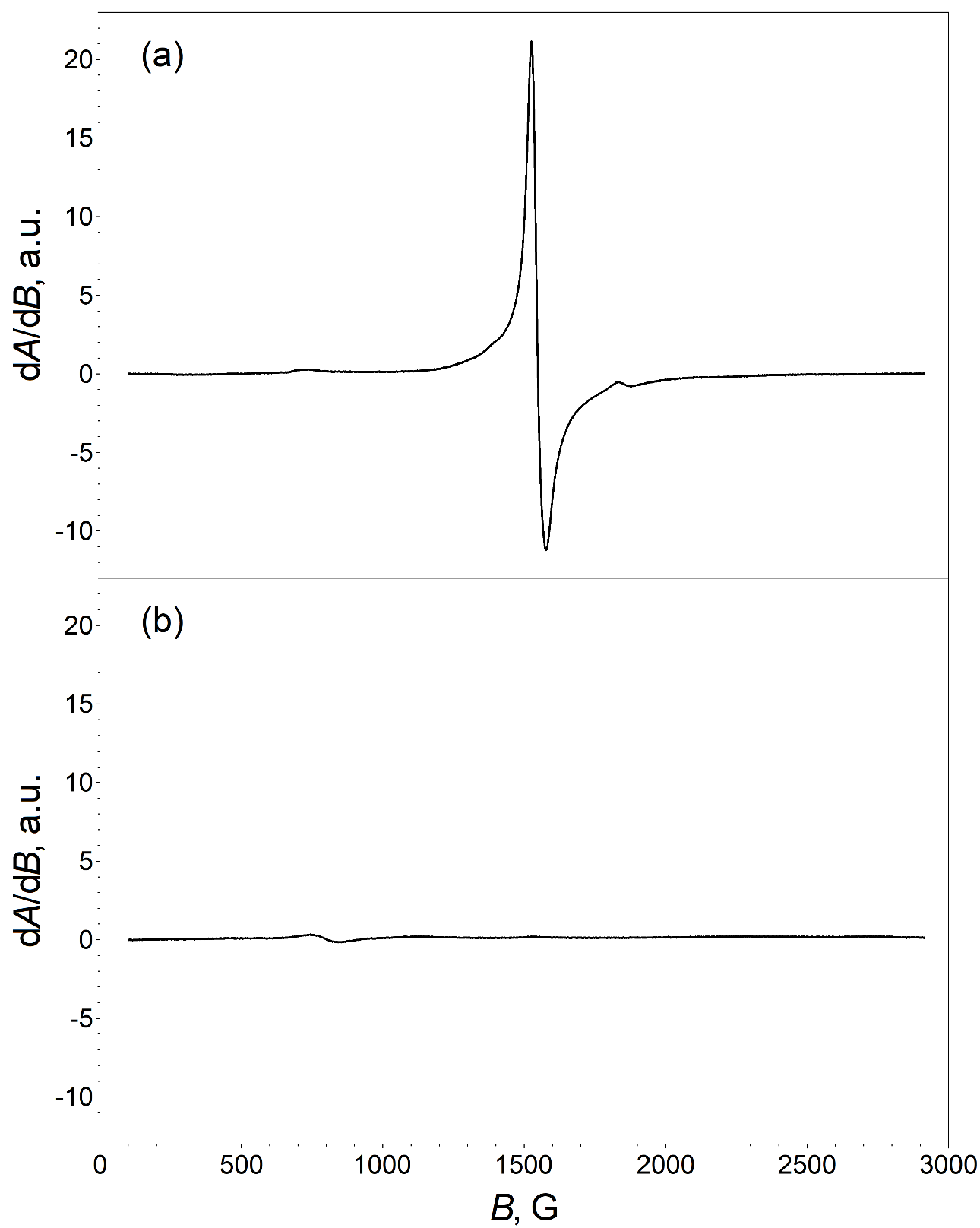


Figure S8. X-band EPR spectra of (a) iron(III) citrate frozen solution with Fe:Cit = 1:100 at pH = 5.5, and of (b) iron(III) citrate frozen solution with Fe:Cit = 1:1 in the presence of water + DMSO mixture. The spectra were measured at 150 K under identical conditions (modulation amplitude of 1 G, microwave power of 2.07 mW).


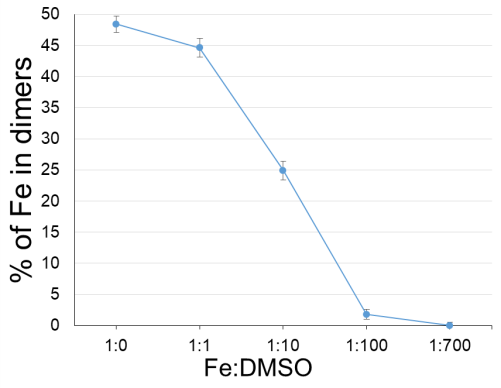

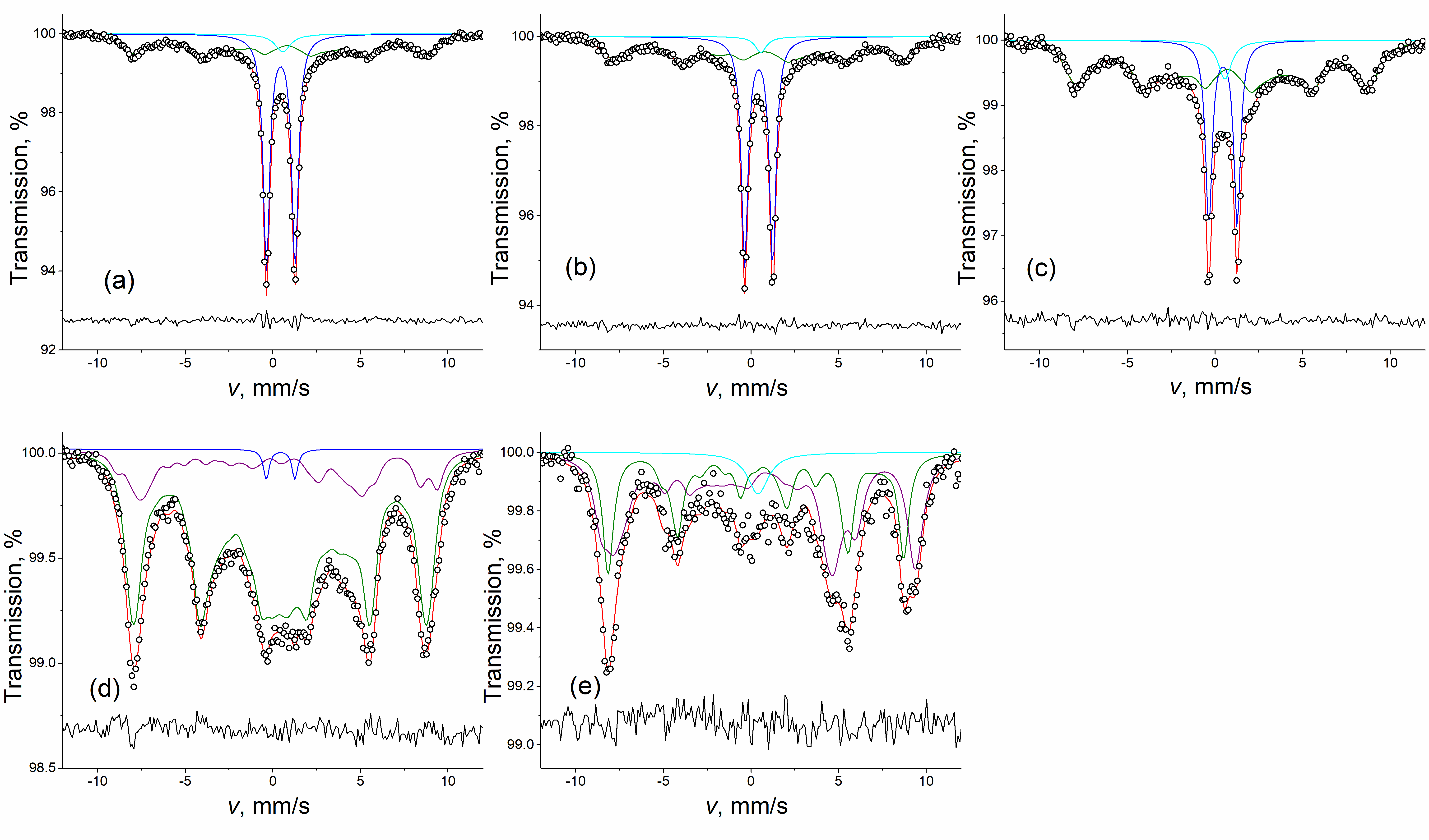
Figure S9. Mössbauer spectra of iron(III) EDTA frozen solutions at different Fe to DMSO ratios: (a) 1:0, (b) 1:1, (c) 1:10, (d) 1:100, (e) 1:700, and the dependence of the fraction of Fe incorporated into dimeric structure from Fe to DMSO ratio
